# Molecular Characterisation of the Haemagglutinin Glycan-Binding Specificity of Egg-Adapted Vaccine Strains of the Pandemic 2009 H1N1 Swine Influenza A Virus 

**DOI:** 10.3390/molecules200610415

**Published:** 2015-06-05

**Authors:** Vincenzo Carbone, Elena K. Schneider, Steve Rockman, Mark Baker, Johnny X. Huang, Chi Ong, Matthew A. Cooper, Elizabeth Yuriev, Jian Li, Tony Velkov

**Affiliations:** 1 AgResearch Limited, Grasslands Research Centre, Tennent Drive, Private Bag 11008, Palmerston North 4442, New Zealand; E-Mail: Vince.Carbone@agresearch.co.nz; 2 Monash Institute of Pharmaceutical Sciences, Monash University, 381 Royal Parade, Parkville 3052, Victoria, Australia; E-Mails: elena.schneider@monash.edu (E.K.S.); elizabeth.yuriev@monash.edu (E.Y.); jian.li@monash.edu (J.L.); 3 CSL Limited Poplar Road, Parkville 3052, Victoria, Australia; E-Mails: Steve.Rockman@biocsl.com.au (S.R.); Chi.Ong@biocsl.com.au (C.O.); 4 Priority Research Centre in Reproductive Science, School of Environmental and Life Sciences, University of Newcastle, Callaghan, NSW 2308, Australia; E-Mail: Mark.Baker@newcastle.edu.au; 5 Institute for Molecular Bioscience, University of Queensland, 306 Carmody Road St Lucia, QLD 4072, Brisbane, Australia; E-Mails: xiao.huang@imb.uq.edu.au (J.X.H.); m.cooper@imb.uq.edu.au (M.A.C.)

**Keywords:** pandemic H1N1 swine influenza A virus, haemagglutinin, glycan binding specificity, egg-adaption

## Abstract

The haemagglutinin (HA) glycan binding selectivity of H1N1 influenza viruses is an important determinant for the host range of the virus and egg-adaption during vaccine production. This study integrates glycan binding data with structure-recognition models to examine the impact of the K123N, D225G and Q226R mutations (as seen in the HA of vaccine strains of the pandemic 2009 H1N1 swine influenza A virus). The glycan-binding selectivity of three A/California/07/09 vaccine production strains, and purified recombinant A/California/07/09 HAs harboring these mutations was examined via a solid-phase ELISA assay. Wild-type A/California/07/09 recombinant HA bound specifically to α2,6-linked sialyl-glycans, with no affinity for the α2,3-linked sialyl-glycans in the array. In contrast, the vaccine virus strains and recombinant HA harboring the Q226R HA mutation displayed a comparable pattern of highly specific binding to α2,3-linked sialyl-glycans, with a negligible affinity for α2,6-linked sialyl-glycans. The D225G A/California/07/09 recombinant HA displayed an enhanced binding affinity for both α2,6- and α2,3-linked sialyl-glycans in the array. Notably its α2,6-glycan affinity was generally higher compared to its α2,3-glycan affinity, which may explain why the double mutant was not naturally selected during egg-adaption of the virus. The K123N mutation which introduces a glycosylation site proximal to the receptor binding site, did not impact the α2,3/α2,6 glycan selectivity, however, it lowered the overall glycan binding affinity of the HA; suggesting glycosylation may interfere with receptor binding. Docking models and ‘per residues’ scoring were employed to provide a structure-recognition rational for the experimental glycan binding data. Collectively, the glycan binding data inform future vaccine design strategies to introduce the D225G or Q226R amino acid substitutions into recombinant H1N1 viruses.

## 1. Introduction

The influenza viruses belong to the family of single-stranded negative sense RNA viruses or Orthomyxoviridae [1,2]. Two surface glycoproteins, haemagglutinin (HA) and neuraminidase (NA) classify influenza A into 18 HA sub-types (H1-H18) and 11 NA subtypes [1,2,3]. Influenza viral infections are responsible for recurrent annual epidemics, and on the order of 250,000 to 500,000 deaths globally [4]. Recurring seasonal epidemics occur because the influenza virus accumulates point mutations in the HA and NA genes during circulation. This process, known as ‘antigenic drift’, allows the virus to escape host immunity. 

The emergence and spread of the novel strain of swine influenza that caused the 2009 H1N1 “swine-flu” pandemic was a matter of strong public health concern [5,6]. The 2009 H1N1 pandemic viruses were first detected in North America around April 2009 and rapidly spread to over 200 countries and have since caused more than 18,000 deaths worldwide [7]. Nonetheless, the majority of these infections were associated with uncomplicated symptomatic upper respiratory tract illness, with a relatively low mortality rate [7]. Phylogenetic analysis of the pandemic H1N1 strains indicated that the genome is composed of six segments derived from a triple reassortant swine H1N1 strain, and two segments from the Eurasian “avian-like” swine H1N1 viruses [8]. The HA gene of the pandemic H1N1 originated from the classical swine influenza lineage which itself is of the human pandemic of 1918-19 viruses ancestry [8]. 

For effective human-to-human droplet transmission, the virus must be capable of efficiently binding to human cell surface receptors and possess the integral proteins that enable it to efficiently replicate in the cells of the human upper respiratory tract. For viral entry, influenza HA binds to sialyl-glycoproteins and sialyl-glycolipids on the surface of host cells [9,10,11]. These sialyl-glycans, usually linked to galactose (Gal) are in either α2,6 or α2,3 configurations. The HA of avian influenza A viruses usually display a preference for α2,3-linked sialyl-glycans, whereas the HA of human influenza A viruses preferentially bind α2,6-linked sialyl-glycans [9,10,11,12,13,14,15,16,17]. The swine viruses have been reported to bind both α2,3- and α2,6-linked sialyl-glycans, but show a greater preference for the latter [9,10,12,13,14,15,16,17]. In the case of the influenza A viruses, the host glycan distribution and binding specificity of the viral HA is a major determinant of the host range of the virus [9,10,11,12,14,15,16,18,19,20,21]. Avian-like α2,3-linked sialyl-glycan receptors can be found on non-coliated cuboidal bronchiolar cells, and alveolar type II cells in the lower respiratory tract. This host glycan distribution might explain why direct human-to-human transmission of avian influenza viruses by coughing and sneezing is inefficient, as the latter would necessitate the presence of avian-type receptors in the upper respiratory tract [22,23,24]. 

The HA receptor-binding site of the prototype 2009 H1N1 pandemic strain A/California/04/09 possesses most of the signature amino acids of human-adapted H1N1 HAs that are required to make optimal contacts with α2,6 sialyl-glycans, in particular D190 on the α-helix and D225 within the 220-loop (H3 numbering used throughout) [25,26]. The receptor binding specificity of representative wild-type pandemic H1N1 2009 viruses, have been characterised directly through co-crystalisation with pentasaccharide sialyl-glycan receptor analogues, using glycan microarray analysis with inactivated viruses and using soluble recombinant HA protein [25,27,28,29,30]. These studies indicated the HA of the 2009 pandemic H1N1 viruses preferentially binds α2,6 sialyl-glycans (human type) [25]. However, glycan array data reported by Childs *et al.* [25] also showed that these viruses also had the ability to bind a range of α2,3 [25] (avian type), albeit with reduced avidity. Egg adaptation of human influenza A viruses often results in a shift in receptor selectivity from an α2,6- to an α2,3-linked sialyl-glycan preference [31]. In light of its α2,6 binding preference, the native A/California H1N1 grows poorly in eggs making vaccine production difficult [32]. We, and others identified mutation sites in the HA molecule that significantly facilitated viral rescue and amplification in eggs namely, K123N, D225G and Q226R [32,33,34]. To understand how these important mutations impact on the receptor binding preference of these key H1N1 2009 vaccine strains, we have now examined the glycan selectivity of each HA variant using a solid-phase ELISA glycan binding assay with both whole viruses and purified recombinant HAs [35,36]. Furthermore, homology-based structural models of the various HA molecules complexed to receptor analogs were constructed to assess amino acid differences within the binding pocket can change HA avidity for human and avian glycans. This study provides the structure-recognition rational for the glycan binding mechanism of important egg-adaption mutations in the HA receptor binding pocket of vaccine production variants of the 2009 H1N1 pandemic influenza A virus. The introduction of these HA mutations into recombinant viruses will enable the rapid generation of future H1N1 vaccine strains with a high growth in eggs.

## 2. Results and Discussion

Initial attempts to obtain a vaccine seed virus by rescue of reverse genetic A/California/07/09 viruses with A/Puerto Rico/8/34 backbone genes proved unsuccessful [34]. This was rectified through the rescue of viruses with the egg-adaption mutations K123N, D225G and Q226R in the viral HA during reassortment [34]. High-throughput sequencing studies of specimens from infected individuals during the second wave of the 2009 H1N1 pandemic in Japan revealed that the K123N, D225G (substitution rate 0.01%–0.11%) and Q226R (substitution rate 0.1%–0.41%) mutations exist as a very minor population in humans [37,38]. The World Health Organisation (WHO) reported that the overall prevalence of the D222G was <1.8% and ~7.1% in fatal cases of H1N1 [39]. Interestingly, Chen *et al.*, [33] reported that egg-adapted strains of A/California/04/09 with Q226R residue and A/California/07/09 with D225G and Q226R HA substitutions reacted similarly to ferret reference sera from animals immunised with the corresponding wild-type strains. In the present study, we examined the receptor binding specificity for egg-adapted vaccine strains of A/California/07/09, and purified recombinant HA proteins with the aforementioned RBS mutations associated with high egg-growth. Furthermore, HA docking models with human and avian receptor analogs were constructed and per residue scoring was performed to provide a structure-recognition rational for the glycan binding data.

### 2.1. Glycan Binding Specificity of Pandemic 2009 H1N1 K123N, D225G and Q226R HA Mutants

**Figure 1:**
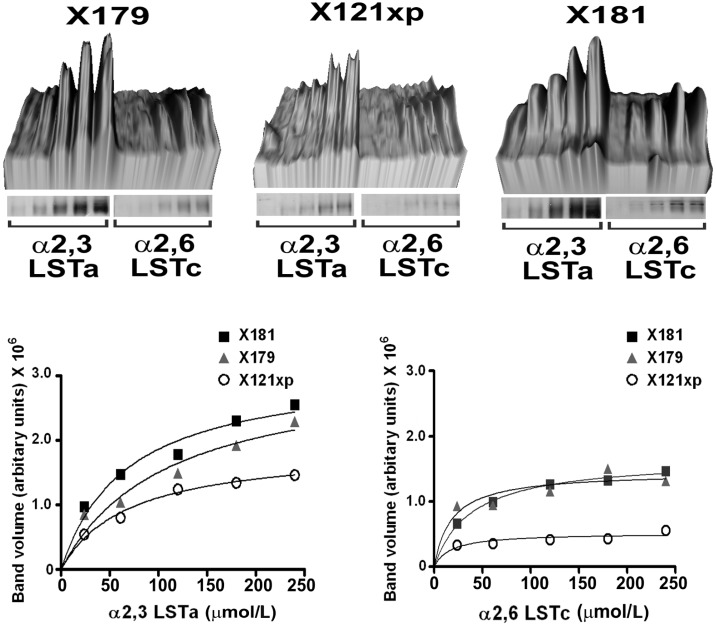
Receptor selectivity of egg-adapted A/California/07/09 H1N1 viruses measured by a whole virus gel-capture binding to resin immobilised human receptor analogs, α2,6-LSTc and the avian receptor analog, α2,3-LSTa. *Top panel.* Densitometric quantification of HA band intensity with increasing sialyl-glycan load. *Bottom panel.* Plots of the captured HA band volume of each virus as a function of the sialyl-glycan load.

The glycan specificity of three egg-adapted A/California/07/09 H1N1 viruses was examined using a glycan gel-capture assay that measures direct binding of the virus particles to resin immobilised LSTa (sialyl-α2,3-galactose, avian receptor analog) or LSTc (sialyl-α2,6-galactose, human receptor analog) (Figure 1). Results revealed that all three viruses display a strong LSTa (α2,3) binding preference with the X181 (N133D, Q226R) virus displaying highest binding affinity which is consistent with its ability to replicate more efficiently in eggs than the other two strains [32]. 

Both the X181 and X179 (Q226R) viruses displayed comparable LSTc (α2,6) binding whereas the X121xp (K123N, Q226R) virus displayed relatively low binding affinity for both LSTa and LSTc. Collectively, the binding data suggests that the LSTa (α2,3) avian receptor binding affinity of these viruses is proportionate to their growth in eggs [32].

The glycan binding specificity of the virus particles and purified recombinant A/California/07/09 HAs harboring the RBS mutations D225G, Q226R, D225G/Q226R and Q226R/K123N was also examined using an expanded glycan array solid phase assays ELISA assay. The glycans in the array are all relevant to influenza biology and are among structures seen in cultured human bronchial epithelial cells [22,40]. The ELISA assay platform was performed in competition format where increasing concentrations of free glycans are used to displace the biotinylated probe glycans (3′-SL or 6′-SL) from which inhibitory dissociation constants are derived (Appendix, Table A1). The ELISA competition data revealed that the X179 (Q226R) and X181 (N136D, Q226R) viruses displayed a comparable pattern of highly specific binding to α2,3-linked sialyl-glycans, with a negligible affinity for most of the α2,6-linked sialyl-glycans. Both viruses displayed a higher affinity for longer *vs.* shorter α2,3-linked sialyl-glycans, as well as for the α1,3 (SLe^X^) and α1,4 (SLe^a^) fructose substituted 3′-sialyl-Lewis glycans. Moderate binding to α2,6-linked biantennary glycans (MSLNH and SLNFPI) was also evident. The X121xp (K123N, Q226R,) virus displayed a comparable glycan binding profile, however, its ability to bind all glycan types was ~5-fold lower than the X179 and X181 viruses. Considering that the X121xp virus also harbors the Q226R mutation, it is possible that the introduction of a glycosylation site through a K123N mutation proximal to the RBS interferes with receptor binding. This is consistent with earlier studies indicating that glycosylation sites proximal to the HA RBS can interfere with receptor binding [41,42,43,44].

The glycan binding data for the purified HA proteins revealed that the native A/California/07/09 HA displays an almost exclusive pattern of binding to α2,6-linked sialyl-glycans, with no binding to most of the α2,3-linked sialyl-glycans in the array (Table A1). This observation is consistent with previously reported glycan array screening data for recombinant A/California/04/09 HA proteins [17,28,45]. Binding to the α2-6-linked biantennary glycans MSLNH and SLNFPI was also evident. The preferred α2,6 *vs.* α2,3-linked sialyl-glycan binding pattern of the native A/California/07/09 HA is consistent with its stronger binding to human upper respiratory tract tracheal tissue sections (that predominantly express α2,6-linked sialyl-glycans) and minimal binding to the lower respiratory tract alveolus (that predominantly express α2,3-linked sialyl-glycans) [29]. These data also support the notion that the A/California/07/09 HA is indeed human adapted.

In the second wave of the 2009 H1N1 pandemic, D225G variants were frequently identified in specimens from the lower respiratory tract, and has been associated with a higher mortality and increased severity of pulmonary infection [38,46,47,48]. The preference for the lower respiratory tract may also account for the lower frequency at which the D225G variant is transmitted [48]. In our hands, the recombinant A/California/07/2009 D225G single mutant HA displayed enhanced binding affinity for both α2,6- and α2,3-linked sialyl-glycans in the array, although its α2,6-glycan affinity was generally higher compared to its α2,3-glycan affinity. In contrast, glycan array analysis using the recombinant HA of the pandemic H1N1 A/Texas/5/09 isolate revealed that the D225G mutation results in a strong α2,6-linked sialyl preference with a weak α2,3-linked sialyl affinity [30]. Glycan array results reported by Lui *et al.*, [49] revealed that an egg-passaged isolate of A/Hamburg/5/09 harboring the D225G HA mutation displayed an enhanced α2,3-glycan affinity and a largely unchanged α2,6-glycan affinity compared to the wild-type virus A/Moldova/G186/09 [49]. This group also noted an overall higher α2,6 *vs.* α2,3 glycan affinity for the D225G mutants [49]. Similarly, Chen *et al.,* [29] reported a dual α2,3 and α2,6 binding specificity for the egg adapted 2009 pandemic H1N1 viruses A/Mexico/Indre/4114/09, and A/New York/04/09 harbouring the D225G HA mutation. More specifically their data showed that these D225G mutants displayed an enhanced affinity for α2,3-linked glycans, whereas the binding to α2,6-linked glycans was comparable to the D225 wild type viruses, A/Ohio/07/09 and A/New York/18/09 [29]. Zhang *et al.*, [45] also demonstrated dual α2,3 and α2,6 binding specificity for D225G recombinant A/California/04/09 HA using a surface Plasmon resonance glycan binding platform to measure the affinity for two biotinylated receptor analogues [45]. Similar to our findings, this group also noted the receptor affinities of the D225G mutant HA were higher than that of the wild type A/California/04/09 HA. Paradoxically, Chen *et al.*, [33] reported a reduced α2,6 binding affinity for egg adapted A/California/04/09 D225G HA mutants using a haemagglutination assay with chicken red blood cells [33]. Similar findings were reported by Takemae *et al.*, [50] who showed egg-adapted D225G mutants of A/Sw/Ratchaburi/NIAH101942/08 and A/Sw/Tochigi/1/08 had an increased haemagglutinating activity for mouse and sheep erythrocytes, that predominantly express α2,3 glycans. Belser *et al.*, [51] also reported a modest reduction in α2,6 glycan affinity of A/California/04/09 D225G recombinant virus with a concomitant increase in α2,3 glycan affinity. A dual α2,3/α2,6 glycan receptor specificity was reported for the A/California/04/09 D225G recombinant virus using a haemagglutination assay with turkey red blood cells [38]. Collectively, these findings are most interesting given that this mutation is associated with both egg-adaption and more sever clinical outcomes. Notably, enhanced α2,3-linked glycan affinity of D225G viruses has been correlated with increased capacity to infect ciliated epithelial cells, alveolar type II pneumocytes, bronchial submucosal glands and Calu-3 bronchial epithelial cells which are prevalent along the entire airway epithelium and perform an important mucociliary clearance functions [38,48,49,52]. Thus the dual receptor specificity of D225G variants may facilitate entry into the lower respiratory tract where α2,3 glycans are more prevalently expressed [22,38,53]. These factors may contribute to the selection of the D225G mutation and to the severity of disease associated with D225G influenza virus pulmonary infections.

In line with the glycan binding data for the Q226R egg adapted viruses, the recombinant A/California/07/09 Q226R single mutant HA displayed a complete loss of α2,6-linked sialyl-glycan affinity with a switch to an α2,3-linked sialyl-glycan preference. Notably, the α2,6→α2,3 switch seen with the Q226R HA single mutant was more pronounced compared to the viruses, as the latter retained some ability to bind to the α2,6-linked biantennary glycans MSLNH and SLNFPI. This drastic loss of α2,6 binding capacity has also been reported for the egg passaged virus A/New York/18/09 harbouring the Q226R HA mutation [29]. Unlike the Q226R single mutation which abolished α2,6 binding, the recombinant A/California/07/09 D225G/Q226R double mutant exhibited a restored capacity to bind α2,6-linked sialyl-glycans, albeit, it still retained the strong α2,3-linked sialyl-glycan preference seen with the Q226R single mutant. This suggests that the latter mutation dominates over the D225G mutation. The recombinant A/California/07/09 K123N/Q226R double mutant HA displayed a pattern of highly specific binding to α2,3-linked sialyl-glycans comparable to the X121xp virus which harbours the same HA mutations, moreover, its ability to bind all of the glycans in the array was ~2–3-fold lower compared to the Q226R and D225G/Q226R recombinant HAs.

Our glycan binding affinity constants are in a similar micromolar range as per previously reported glycan binding data garnered for recombinant A/California/04/09 HAs using a Surface Plasmon Resonance assay platform [45]. Notably, we observed that virus particles displayed an overall higher receptor binding affinity when compared to the recombinant HA proteins, presumably because the higher density of trimeric HA protein on the virion surface acts to enhance glycan binding. This increased valency may also account for the dual α2,3/α2,6 binding reported by Childs *et al.*, [25] for glycan array experiments with BPL inactivated pandemic 2009 H1N1 virus isolates. However, this discrepancy may also be due to the type of glycan array platform used as two other groups reported a strict α2,6 glycan preference for pandemic 2009 H1N1 viruses [27,29]; consistent with our binding data for the wild-type recombinant A/California/07/09 HA. On this note, the discrepancies in the literature noted above for the D225G variants may also be a result of the type of glycan binding platform employed and again the use of whole virus particles *versus* purified HA protein.

### 2.2. Glycan Structure-Recognition Characteristics of Pandemic 2009 H1N1 D225G, Q226R and D225G/Q226R HA Mutants Examined through in Silico Docking and Modeling

Our *in silico* docking results with the wild-type A/California/07/09 HA depicted what has previously been well-established, that is a preference for the α2,6-linked sialyl pentasaccharide LSTc over the α2,3- LSTa (Appendix, Table A2). The LSTc-HA interactions seen in our docking model were consistent with the interactions seen in the reported co-crystallographic structure [17]; interactions between LSTc and RBS residues Y98, K222, T136, H180, D225, Q226. With the modeled α2,3 LSTa pentasaccharide we observed interactions with RBS residues Y98, V135, T136, A137, H180, S186, D190 and Q226. A clear distinction between the binding modes can be made by the enumerable hydrogen bond contacts between the 190-helical domain of the RBS and the terminal glycan moieties of LSTc (α2,6); which are absent in the bound configurations of LSTa (α2,3) (Figure 1A).

**Figure 2:**
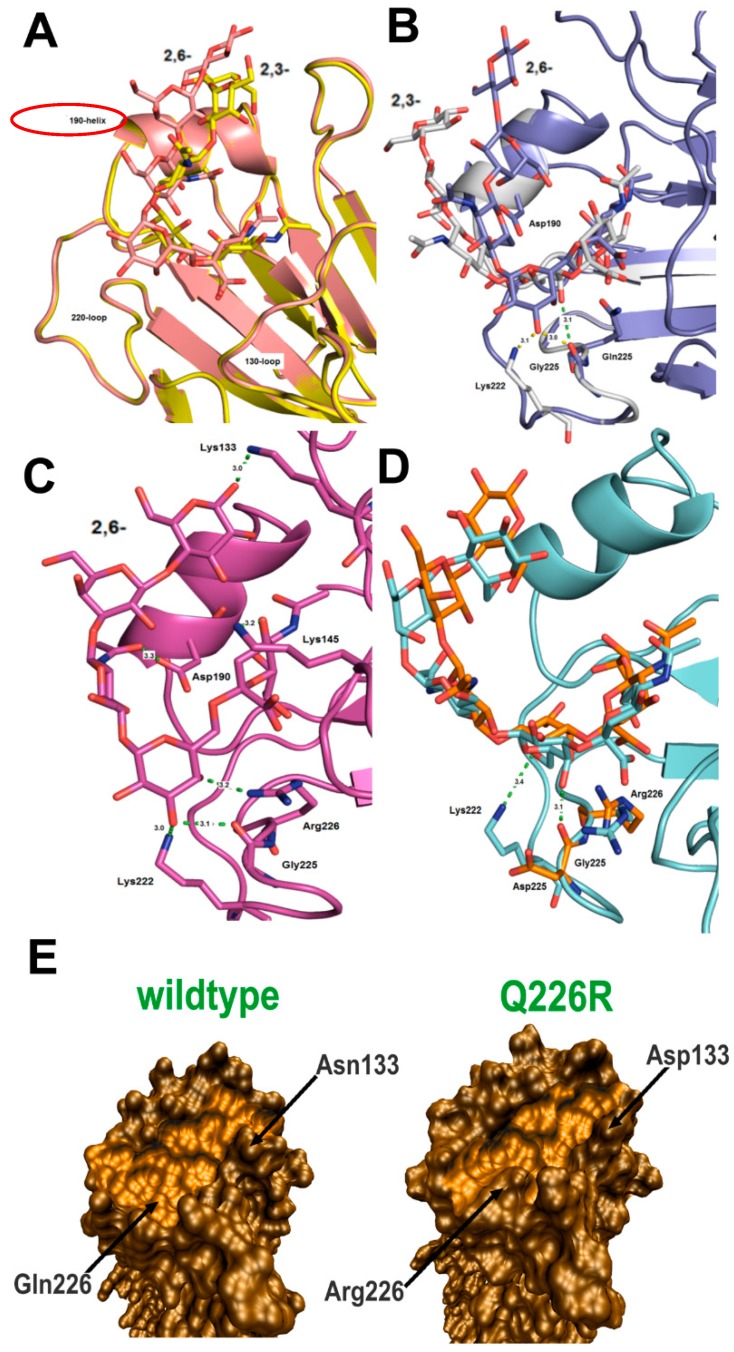
(**A**) Docked and superimposed confirmations of LSTc (α2,6) & LSTa (α2,3) in the RBS of A/California/07/09 HA and in the mutated models; (**B**) The D225G mutant with the docked LSTc (α2,6) & LSTa (α2,3); (**C**) LSTc (α2,6) docked in the D225G/Q226R double mutant; and (**D**) Q226R (in orange) superimposed on the D225G/Q226R (in blue) mutant and the docked LSTc (α2,6). Hydrogen bonds are in dotted lines. Distances are given in angstrom; (**E**) Solvent-excluded surfaces for each structure calculated with a 1.4 Å probe. The receptor-binding surface of the cavity is lightly shaded. The binding cavity of the Q226R is narrower than the wild type A/California/07/09 pocket making it more suited to accommodating the extended conformation of the LSTa (α2,3) avian receptor analog.

The D225G mutant produced interesting results, with an increase in binding values for both LSTc and LSTa (Figure 2B). Zhang *et al.*, [45] have previously established that the D225G single mutation switches binding specificity to a dual receptor specificity, and Pan *et al.*, [54] demonstrated a free energy gain for the α2,3- glycans in a D225G mutant of H1N1 brought about by an increase in energy contribution by Q226. These observations correlate well with our own *in silico* results where LSTa (α2,3) possess a fitness value similar to LSTc (α2,6) in the wild type and we observe a large increased score for Q226 in the model (Table A2). The Gal2 moiety of LSTa (α2,3) sits lower within the RBS, (presumably due to the absence of side chain at the 225 position) and forms a hydrogen bond contact with the carbonyl of glycine with the hydroxyl of Gal2 (3.1 Å). Additional hydrogen bond contacts are also observed with S186, T187, D190 and Q192, and the remaining sugar moieties of LSTa (α2,3) (Figure 2B).The disestablishment of a salt bridge between K225 and D225 may also be responsible for the increase in binding values seen for LSTa (α2,3) with both residues G225 and K222 showing an increase in per atom scoring. A similar increase in binding values are seen with the LSTc (α2,6) model as are hydrogen bond contacts with the side chain amine of K222 (NZ: 3.1 Å) and carbonyl of G225 (O; 3.0 Å) with the hydroxyl of Gal2 (Table A2).

The docking model for the Q226R mutant depicts a near halving of the affinity of LSTc (α2,6) receptor suggesting that the molecule does not bind efficiently, if at all (Figure 2C and Table A2). Simple superimposition can place rotamer states of the larger R226 side chain within 1.8–2.2 Å of the sialic acid and Gal2 moieties, suggesting large movements would be required of the receptor or potentially unfavorable rotameric states of the arginine side chain would be necessary to accommodate LSTc (α2,6). In the Q226R model we observe far fewer hydrogen bond contacts with the 220-loop and the 190-helix and D225 scoring suggest a potential clash with Gal2. Conformational analysis has also shown that that LSTc (α2,6) displays a greater range of conformations in solution compared to the LSTa (α2,3) [19]. Thus, the larger binding pocket of human H1 HAs more favorably accommodates the binding of the more flexible α2,6 configuration. The Q226R substitution, however, makes the right corner trough of the binding pocket narrower compared to the binding pocket of wild-type A/California/07/09 HA (Figure 2E). This makes the RBS cavity more suited to accommodating the extended conformation of the α2,3-linked avian receptor analog LSTa. This is consistent with the reduced width of the receptor binding pocket seen in the avian H5 viruses that enhances the interaction with α2,3-receptors [16,51].

LSTc (α2,6) binding, however, is rescued in the D225G/Q226R double mutant HA model, where we notice positive residue scoring at those positions. *In silico*, G225 affords R226 far more energy favorable rotameric states to accommodate α2,6- receptor and no potential clashes with Gal2 as it lacks a side chain. G225 could also produce added flexibility to the 220-loop not afforded by D225. Hydrogen bond interactions within the LSTc (α2,6)-D225G/Q226R double mutant complex of note include K133 (NZ) and the hydroxyl group on the terminal Gal moiety (3.0 Å), D190 side chain (OD1) and GlcNac3 (3.0 Å), K145 (NZ) and the hydroxyl present on sialic acid (3.2 Å), the hydroxyls of Gal2 with the side chain amines of K222 (NZ; 3.0 Å), R226 (NH1; 3.2 Å) and the carbonyl of G225 (O; 3.1 Å) (Figure 2D). These interactions are exclusive to LSTc (α2,6) and do not affect LSTa (α2,3) binding regardless of mutation which produced near consistent scoring throughout the docking process (Table A2). In fact point mutations only increased scoring in those same positions, with hydrogen bond contacts occurring between the hydroxyls of Gal2 and the carbonyl of G226 (O; 3.1 Å) and K225 (Figure 1D; O; 3.4 Å). 

## 3. Experimental Section 

### 3.1. Materials

Receptor analogs Neu5A, 3′-SL, 6′-SL, 6′-SLN, DSLNT, LSTa, LSTb, LSTc, 3′-SL-ADP-HSA, 6′-SL-ADP-HSA, SLe^a^, SLe^X^, Le^a^, SLNFPI, MSLNH, and LST-fractogels (Table A1) were either from IsoSep AB (Tullinge, Sweden) or Sigma-Aldrich (Melbourne, Australia). Biotinylated 3′-SL and 6′-SLN were obtained from Glycotech (Gaithersburg, MD, USA). Nunc immobiliser 96 well-plates were obtained from Thermo Fisher Scientific (Melbourne, Australia). All materials and chemicals were of the highest commercial quality available.

### 3.2. Viruses

The introduction of egg-adaption mutations into the viral HA and rescue of reverse genetic A/California/07/09 viruses with A/Puerto Rico/8/34 backbone genes in Vero cells was performed as previously described [34]. Viruses were propagated by growth for 48 h in the allantoic cavities of 10-day-old embryonated chicken eggs at 37 °C and infectious allantoic fluids were harvested and pooled. All viruses were purified by rate zonal centrifugation and inactivated by β-propiolactone (BPL) (0.05% *v*/*v*) treatment at 4 °C. BPL was deactivated by hydrolysis at 37 °C overnight. BPL inactivation has previously been shown not to affect HA receptor-binding properties of influenza viruses [25,49]. Viruses were quantified by the haemagglutination assay [49,55]. Haemagglutination titers were determined by standard procedure using chicken red blood cells in phosphate buffered saline solution (PBS) [55].

### 3.3. H1N1 HA Protein Expression and Purification

The ectodomain (HA1 residues 11 to 329 and HA2 residues 1 to 174 [H3 numbering]) of the A/California/07/09 HA and the RBS mutants were expressed in Sf9 cells after being cloned into a pFastBac1 vector encoding a *N*-terminal gp67 signal peptide for secretion, trimerisation foldon and a C-terminal thrombin cleavage site and [His]_6_ purification tag. The D225G, Q226R, D225G/Q226R and K123N/Q226R RBS mutants were constructed by site-directed mutagenesis using the QuikChange II site-directed mutagenesis kit (Agilent Technologies, Mulgrave, Australia). The transfection and virus amplification steps were performed as per the manufacturer’s instructions for the Bac-to-Bac baculovirus expression system (Invitrogen, Melbourne, Australia). About 48 h post infection the cell culture supernatant was buffer exchanged and concentrated into immobilised metal ion affinity chromatography (IMAC) binding buffer (20 mM Tris-HCl pH 8.0, 50 mM NaCl) and imidazole added to a final concentration of 20 mM before loading to a IMAC column (5 mL HisTrap FF, GE HealthCare, Melbourne, Australia). Unbound proteins were removed with binding buffer for 20 column volumes (CV) before elution with a gradient to 50% elution buffer (20 mM Tris-HCl pH 8.0, 50 mM NaCl, 500 mM imidazole) over 10 CVs, to 100% B over 2 CVs, then hold at 100% B for a further 10 CVs. The HA containing fractions were pooled and dialysed against binding. The HA was then further purified by ion-exchange chromatography on a Mono-Q anion exchanger column (GE HealthCare, Melbourne, Australia). Pooled HA containing fractions were subjected to thrombin digestion, 3 U/mg HA 12 h at 4 °C. Cleavage reactions were subsequently loaded onto Superdex 200 prep grade Hiload 26/60 size-exclusion chromatography column (GE HealthCare) and resolved at a flow rate of 2 mL/min using 20 mM Tris-HCl pH 8.0, 50 mM NaCl buffer. Fractions of the purified HA were collected and sampled for SDS-PAGE before pooling them together.

### 3.4. Gel-Capture Assay 

LSTa and LSTc fractogels were synthesised by IsoSep AB as described by [56]. Fractogel TSK HW65 (F) (Toyopearl) was obtained from Merck (Kenilworth, NJ, USA). Derivatisation to an amino-form was performed by the treatment with epichlorohydrine and subsequent opening of the epoxide with ammonia [57]. The gel in the amino-form (1 g wet weight) was mixed with the reducing oligosaccharide LSTa or LSTc (7–14 µmol) and 3 mL methanol and allowed to react for 10 h at 60 °C, what resulted in the corresponding glycosyl-amine. Approximately 1 mL of the mixture in acetic anhydride was kept for 12 h at 20 °C to give the corresponding glycosylamide. BPL inactivated viruses in PBS were standardised to 150 μg/mL HA protein and 100 μL aliquots were incubated with 2–300 μL (400 µmol/L) of either LSTa or LSTc gel slurry (approximately 2.0 μmol/g substitution, determined by weight of oligosaccharide after substitution) at 4 °C for 1 h. The total reaction volumes were 400 µL in PBS buffer. The sialidase inhibitor oseltamivir (10 μmol/L) was included in all incubations. The gel slurry was sedimented by centrifugation at 6000× *g* for 1 min and washed four times with 300 μL PBS. Captured virus was released with 100 μL SDS-PAGE sample buffer and approximately 10 μL were resolved on 4%–20% gradient polyacrylamide gel. The gels were silver or Coomasie blue stained to visualise proteins, dried, and scanned at 1230 dpi. HA protein bands were quantified densitometrically using LabImage 1D gel analysis software V3.4 (Kapelan Bio-Imaging GmbH, Leipzig, Germany). 

### 3.5. ELISA Solid-Phase Assay

This procedure was performed using the biotinylated 3′-SL or 6′-SL, essentially as previously described with minor modifications to the original protocol [40,58]. In brief, Nunc immobiliser 96 well-plates were coated with influenza virus (0.1 mg/mL HA) and blocked with 1% BSA. Various dilutions of biotinylated sialyl-glycans at the desired concentrations in PBS were added into the wells (50 µL/well) and the plates were incubated at 4 °C for 1 h. For the competition experiments, increasing concentrations of free receptor analogs were added to the incubation mixture containing a saturating concentration (5 µM) of biotinylated 3′-SL or 6′-SLN. To inhibit viral sialidase activity, oseltamivir (10 µM) was included in all wells. The wells were washed four times with PBS and bound biotinylated sialyl-glycans were quantified with 25 µL/well of HRP-streptavidin (1:2000). Plates were incubated at 4 °C for 1 h. After washing with PBS, the peroxidase activity was assayed with *O*-phenylenediamine solution and the reaction was stopped with 50 µL of 1M HCl. Absorbance was determined at 490 nm. The apparent inhibition constant (K_i_) for virus binding to the displacing glycan was determined from the non-linear regression fit of the plot of the displacing glycan concentration *vs.* the A_490_ to a one-site-competition model from which EC_50_ values were derived. The inhibition constant (K_i_) for glycan binding was calculated according to the equation K_i_ = EC_50_/(1 + [biotinylated sialyl-glycan]/K_D_ biotinylated sialyl-glycan). All data modelling operations were performed using GraphPad Prism V4.0 software (GraphPad software, San Diego, CA, USA).

### 3.6. Homology Modeling of the HA Structure and Receptor Selectivity

Molecular docking experiments were carried out using the program Genetic Optimisation for Ligand Docking (GOLD), version 5.2 [59]. All docking runs where performed on the Influenza haemagglutinin (H1N1) from the 2009 pandemic H1N1 in complex with ligand LSTc (PDB code 3UBE) [17]. Utilising the macromolecular model building program Coot [60] mutants of 3UBE where built including D225G, Q226R and D225G/Q226R. For each GOLD run the binding site was composed of all residues that fell within 6 Å the bound LSTc. To ensure common binding motifs of the human receptor analog LSTc (α2,6 sialyl-glycan) and avian receptor analog LSTa (α2,3 sialyl-glycan) hydrogen bond interactions where promoted between the o-sialic acid moiety and the receptor binding site residues. Side-chain residues were rigid with the exception of the Q226R mutants, where a number of potential rotamers of R226 where described and included in the docking run. No water molecules were defined as part of the active site. GOLD runs were carried out using automatic GA settings of 10 and with 100% search efficiency. A Piecewise Linear Potential (PLP) scoring option was the preferred function using default parameters. Per atom scoring was enabled and tracked to best observe the steric complementarity between protein residues and ligand. The solvent excluded surfaces of the receptor binding sites were calculated with the program MSMS [61] and generated with VMD [62] and PovRay (V3.62). All other structures were generated with PyMol [63].

## 4. Conclusions 

The emergence of future influenza virus pandemic strains from swine was only a matter of time. Seasonal vaccination allows our immune system to develop antibodies against the HA and NA, thereby providing the human population with a level of protective immunity against similar strains. The efficient generation and egg propagation of H1N1 influenza virus strains is crucial for vaccine production. The production of a 2009 H1N1 influenza vaccine seed strain capable of efficient replication in eggs, whilst maintaining the appropriate antigenicity and immunogenicity, was initially a challenging task. Our glycan binding data support the introduction of the D225G or Q226R mutations in recombinant H1N1 viruses to achieve high egg-growth to for rapid generation of vaccine production candidates. 

## Abbreviations

Neu5A: *N*-Acetylneuraminic acid
3′-SL: 3′-sialyl(*N*-acetyllactose)
3′-SLN: 3′-sialyl(*N*-acetyllactoseamine)
6′-SL: 6′-sialyl(*N*-acetyllactose)
6′-SLN: 6′-sialyl(*N*-acetyllactoseamine)
BPL: β-propiolactone
DSLNT: Disialyllacto-*N*-tetraose
HA: haemagglutinin
Le^a^: Lewis sugar A
LSTa: sialyllactose-*N*-tetraose a
LSTb: *N*-Acetylneuramin-lacto-*N*-tetraose b
LSTc: sialyllactose-*N*-tetraose c
MSLNH: Monosialyllacto-*N*-hexaose
SLe^a^: 3′-Sialyl-Lewis-a tetrasaccharide
SLe^X^: 3′-Sialyl-Lewis-X-tetrasaccharide
SLNFPI: Sialyllacto-*N*-fucopentaose I

## Appendix

**Table A1:** Glycan binding selectivity of A/California/07/09 vaccine strains viruses and recombinant A/California/07/09 HAs.

Glycan	Ki (µM)							
	X179 Virus Q226R	X121xp Virus Q226R, K123N	X181 Virus Q226R	A/Cal /07 HA Native	A/Cal /07 HA D225G	A/Cal /07 HA Q226R	A/Cal /07 HA D225G, Q226R	A/Cal /07 HA Q226R, K123N
***N*-Acetylneuraminic acid (Neu5Ac)** Neu5Ac	^1^ NB	^1^ NB	^1^ NB	^1^ NB	^1^ NB	^1^ NB	^1^ NB	^1^ NB
**3′-Sialyl(*N*-acetyllactose)(3′-SL)** Neu5Acα2-3Galβ1-4Glc	0.8 ± 0.3	3.0 ± 0.4	1.1 ± 0.2	^1^ NB	2.6 ± 0.4	1.8 ± 0.7	2.1 ± 0.2	4.4 ± 0.9
**3′-Sialyl(*N* -acetyllactoseamine) (3′SLN)** Neu5Acα2-3Galβ1-4GlcNAc	0.6 ± 0.1	2.4 ± 0.5	0.5 ± 0.1	^1^ NB	2.0 ± 0.4	1.6 ± 0.3	1.5 ± 0.3	3.8 ± 0.4
**6′-Sialyl(*N*-acetyllactose) (6′-SL)** Neu5Acα2-6Galβ1-4Glc	^1^ NB	^1^ NB	^1^ NB	3.4 ± 0.7	1.4 ± 0.4	^1^ NB	3.6 ± 0.7	^1^ NB
**6′-Sialyl(*N*-acetyllactoseamine) (6′SLN)** Neu5Acα2-6Galβ1-4GlcNAc	^1^ NB	^1^ NB	^1^ NB	3.1 ± 0.6	0.7 ± 0.3	^1^ NB	2.7 ± 0.4	^1^ NB
**Disialyl-lacto-*N*-tetraose (DSLNT)**	0.5 ± 0.2	2.6 ± 0.4	1.0 ± 0.4	6.9 ± 2.1	3.7 ± 1.0	1.7 ± 0.6	2.5 ± 0.5	3.3 ± 0.2
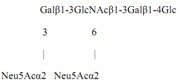
**Sialyllactose-*N*-tetraose a (LSTa) **	0.3 ± 0.1	1.4 ± 0.5	0.5 ± 0.2	^1^ NB	2.7 ± 0.8	0.9 ± 0.2	1.8 ± 0.6	2.4 ± 0.7
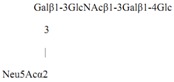
***N*-Acetylneuramin-lacto-*N*-tetraose b (LSTb) **	^1^ NB	^1^ NB	^1^ NB	12 ± 2.0	15 ± 3.9	^1^ NB	^1^ NB	^1^ NB
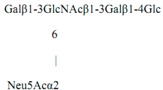
***Sialyllactose-N-tetraose c (LSTc) ***	16 ± 3.5	^1^ NB	14 ± 2.7	2.8 ± 0.4	0.6 ± 0.2	^1^ NB	2.1 ± 0.3	^1^ NB
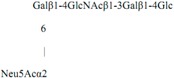
***3′-Sialyl-Lewis-a tetrasaccharide (SLea) ***	0.9 ± 0.1	2.3 ± 0.6	1.1 ± 0.4	10 ± 2.6	3.1 ± 1.0	1.3 ± 0.1	1.7 ± 0.4	3.1 ± 0.4
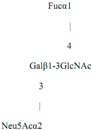
**3′-Sialyl-Lewis-x tetrasaccharide (SLe^x^)**	0.6 ± 0.2	4.1 ± 1.2	0.4 ± 0.1	13 ± 3.9	4.2 ± 1.4	2.1 ± 0.2	3.0 ± 0.6	6.3 ± 2.2
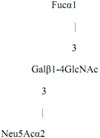
**Lewis sugar A (Le^a^) **	8.1 ± 1.6	16 ± 3.1	10 ± 2.0	^1^ NB	^1^ NB	16 ± 3.1	^1^ NB	^1^ NB
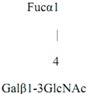
**Sialyllacto-*N*-fucopentaose I (SLNFPI) **	14 ± 2.0	^1^ NB	12 ± 1.5	8.8 ± 2.5	4.5 ± 1.2	^1^ NB	8.8 ± 1.6	^1^ NB
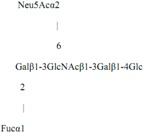
**Monosialyllacto-*N*-hexaose (MSLNH) **	9.8 ± 2.9	^1^ NB	13 ± 3.2	4.2 ± 0.5	2.4 ± 0.8	^1^ NB	5.3 ± 1.6	^1^ NB
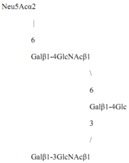

^1^ NB: No binding detected.

**Table A2:** Individual PLP fitness scores for A/California/07/09 HA receptor binding site (RBS) residues and their interaction with LSTc (α2,6) and LSTa (α2,3) following *in silico* docking with Gold.

		Receptor	PLP Fitness Score			
			H1N1 Wild Type	D225G Single Mutant	Q226R Single Mutant	D225G/Q226R Double Mutant
**Total Score**		**α2,6-**	**69.7**	**83.5**	**37.4 **	**63.1 **
		**α*2*,*3*-**	** 56.3**	** 66.9**	**61.4**	**59.9**
**Residues of Note within the RBS**	**Tyr98 **	**α2,6-**	3.97	6.62	6.44	6.76
		**α*2*,*3-***	5.73	7	3.02	6.86
	**His183**	**α2,6-**	2.65	5.47	1.69	4.94
		**α *2*,*3-***	0.82	3.02	3.39	4.85
	**Trp153**	**α2,6-**	1.47	11.25	4.2	9.36
		** α*2*,*3-***	4.24	−2.72	7.44	8.73
	**Ala189**	**α2,6- **	7.55	0.04	4.96	0.13
		**α*2*,*3-***	-	5.11	4.66	0.50
	**Asp190**	**α2,6- **	6.25	10.31	0.57	1.15
		**α*2*,*3-***	6.18	6.75	14.84	4.96
	**Lys133**	**α2,6-**	0.65	0.26	0.12	−0.28
		**α*2*,*3-***	−0.97	0.71	1.27	−1.59
	**Val135**	**α2,6- **	0.51	1.68	0.87	6.99
		**α*2*,*3-***	6.85	7.39	4.21	6.26
	**Thr136**	**α2,6- **	6.84	6.89	6.39	8.16
		**α*2*,*3-***	5.45	9.43	4.66	8.61
	**Ala137**	**α2,6-**	5.71	5.25	5.82	5.21
		**α*2*,*3-***	5.13	2.25	0.03	5
	**Ala138**	**α2,6-**	0.14	0.04	0.08	0.07
		**α*2,3-***	0.08	0.17	0.01	0.08
	**Lys145**	**α2,6- **	1.24	2.72	6.97	6.53
		**α*2*,*3-***	1.05	7.76	0.66	6.23
	**Val155**	**α2,6-**	0.36	0.97	0.46	1.43
		**α*2*,*3-***	-	-	1.15	0.45
	**Lys156**	**α2,6-**	0.02	-	-	0.51
		** α*2*,*3-***	1.38	-	-	-
	**Ser186**	**α2,6-**	0.05	0.06	-	-
		**α*2*,*3-***	4.7	4.64	4.74	0.03
	**Ser193**	**α2,6-**	8.63	4.94	3.18	4.87
		** α*2*,*3-***	7.34	0.43	2.87	1.82
	**Gln192**	**α2,6-**	4.58	0.92	0.94	-
		** α*2*,*3-***	4.81	3.55	0.18	-
	**Leu194**	**α2,6-**	3.61	4.01	2.32	4.27
		** α*2*,*3-***	1.63	0.68	5.09	2.36
	**Lys222**	**α2,6-**	4.20	5.32	2.55	2.26
		** α*2*,*3-***	-	0.09	0.06	0.51
	**Glu227**	**α2,6-**	0.07	-	-	-
		**α*2*,*3-***	0.71	1.01	1.65	1.92
	**Asp225**	**α2,6- **	10.11	N/A	−0.95	N/A
		**α*2*,*3-***	0.14	N/A	0.15	N/A
	**Gln226**	**α2,6- **	9.68	9.25	N/A	N/A
		**α*2*,*3-***	8.36	12.3	N/A	N/A
	**Asp225Gly**	**α2,6- **	N/A	8.88	N/A	8.15
		**α*2*,*3-***	N/A	1.31	N/A	3.92
**Mutated Residues**	**Gln226Arg**	**α2,6- **	N/A	N/A	9.71	7.15
		**α*2*,*3-***	N/A	N/A	12.96	19.12

N/A Not accessed.
